# Visual and refractive outcome, higher order aberrations and complications following intraocular lens exchange in eyes without comorbidities

**DOI:** 10.1038/s41598-025-98931-y

**Published:** 2025-04-23

**Authors:** Nikola Henningsen, Ramin Khoramnia, Gerd U. Auffarth, Maximilian K. Köppe, Grzegorz Łabuz, Timur M. Yildirim

**Affiliations:** Department of Ophthalmology, The David J Apple Center for Vision Research, University of Heidelberg, Im Neuenheimer Feld 400, 69120 Heidelberg, Germany

**Keywords:** Medical research, Outcomes research

## Abstract

Intraocular lens (IOL) exchange is performed to treat severe IOL-related complications. There is little published data on the impact of this procedure on the refractive outcomes and intra- and postoperative complications, especially in otherwise healthy eyes. We investigated the refractive outcome of IOL exchange surgery, including corneal aberrations; additionally, we assessed the influence of different IOL characteristics on intraoperative and postoperative complications. This prospective clinical study included 35 eyes with homogenous IOL calcification without other ocular pathologies. Using Pentacam AXL Wave (Oculus Optikgeräte GmbH, Wetzlar, Germany), corrected distance visual acuity (CDVA), target refraction compared to the postoperative spherical equivalent, corneal topography and lower and higher-order aberrations were assessed preoperatively and from 3 months after surgery. Intraoperative and postoperative complications were recorded and compared between different IOL characteristics. The secondary IOL in 53% of cases, was a retropupillary iris-fixated Artisan Aphakia (Ophtec BV, Groningen, Netherlands), 37% had a sulcus-fixated AR40e (Johnson & Johnson Vision, Irvine, USA), and 10% had a capsular bag IOL. The CDVA improved from 0.16 ± 0.14 to 0.07 ± 0.14 logMAR (*p* = 0.04). In most cases, the target refraction was within ± 1.0 D (Artisan: 71%, AR40e: 90%, Capsular: 100%). IOL exchange did not induce relevant change in corneal aberrations. Anterior (81%) and posterior (78%) vitrectomy were performed in most cases. The haptic design of the primary IOL did not impact intra- or postoperative complications. Although exchanging an IOL involves greater surgery compared to the initial IOL implantation, visual and refractive outcomes are good, and the exchange does not cause relevant change in aberrations. Intra- and postoperative complications are mostly mild and resolve without sequelae.

## Introduction

Intraocular lens (IOL) exchange is the only treatment option for some pathologies like IOL dislocation or IOL calcification^1^. Especially in otherwise healthy eyes, it is essential to minimize intra- and postoperative complications and keep the surgery’s impact on the refractive outcome to a minimum. Intraoperative complications include zonular dehiscence, insufficient capsular bag support as well as the need for vitrectomy^2,3^. Studies on IOL exchange have reported that during the first year after IOL exchange about one-fifth of the eyes require one or more revision surgeries: to address dislocation of the secondary IOL, retinal detachment, epiretinal membrane or pupillary block^3,4^. The authors emphasize the high potential risks of IOL exchange even in healthy eyes^3^. If the capsular bag cannot be preserved, the secondary IOL has to be placed differently, e.g. in the ciliary sulcus^2^ or fixated to the sclera or iris^3^. Concerning the refractive outcome, studies show that secondary IOLs reach deviations of the absolute refractive error from the estimated refractive error of below 1 diopter (D)^4^. Increasing corneal astigmatism and inducing corneal or total higher order aberrations should be avoided. Just a few studies have analysed the surgically induced astigmatism (SIA) following IOL exchange^5,6^. Eum et al. reported an increased SIA following IOL exchange compared to IOL refixation for IOL dislocation^5^. Kubaloglu et al. described a slightly higher SIA after IOL exchange using a clear corneal incision and cutting the IOL in half for explantation compared to standard cataract surgery^6^. Little is known about the impact of IOL exchange on higher order aberrations.

This study aims to analyse the refractive outcome, intra- and postoperative events of IOL exchange using different secondary IOL fixation sites in eyes with primary IOL calcification alone and without confounding comorbidities. Furthermore, the effects of IOL exchange on corneal aberrations of lower and higher order are evaluated.

## Material and methods

### Patient enrolment

This prospective clinical study included patients scheduled for IOL exchange due to monocular or binocular homogenous IOL calcification. The patients had to be over 18 years of age and give written informed consent before participating in this study. Exclusion criteria were any other ocular comorbidities, including corneal dystrophy, known zonula instabilities, glaucoma or retinal impairments as well as neurological conditions such as dementia, pregnancy or any other systemic medical conditions that could adversely affect the outcome. The study was completed in accordance with the principles of the Declaration of Helsinki. Prior to the study, Institutional Review Board approval was obtained from the local Ethics Committee (S-193/2022).

### Surgery technique

One of two experienced surgeons (GUA and RK) exchanged the primary for a secondary IOL. The secondary IOLs were calculated using the SRK-T formula. In all cases, the incisions from the first and the second surgery were created at different locations. For the IOL exchange, two 20-gauge paracentesis incisions and a superior sclero-corneal tunnel with an incision size of 5 mm were created. Next, the calcified IOL was mobilized out of the capsular bag into the anterior chamber and removed via the sclero-corneal tunnel. If needed and depending on the conditions of the intraocular structures, the surgeon performed an anterior and/ or posterior vitrectomy. The secondary IOL was implanted through the scleral-corneal tunnel. Depending on the type of the lens, it was placed in the capsular bag, in the ciliary sulcus, or, in the absence of sufficient capsular support, an iris-claw-lens was enclaved horizontally behind the iris. The surgery was completed with a scleral cross-suture, a conjunctival suture and injection of intracameral antibiotics.

### Outcome measurements

Patients were examined one day before and after surgery, and once from three months after IOL exchange. Additional visits were staged as needed. Information about the calcified IOL were obtained from the patients’ records. Intra- and postoperative events were collected from the intraoperatively until to the final study visit. At each study visit, a subjective refraction was conducted using Early Treatment Diabetic Retinopathy Study (EDTRS) charts to calculate the spherical equivalent and to obtain the corrected distance visual acuity (CDVA) prior to ocular examination including intraocular pressure (IOP) measurement using Goldmann applanation tonometry and slit lamp examination. The preoperative target refraction was assessed using the IOLMaster 700 (Carl Zeiss Meditec AG, Oberkochen, Germany). In five eyes, due to the severity of the IOL opacity, the axial length could not be obtained using the IOLMaster 700, and A-scan biometry was used instead, in one case the measurements were taken from an earlier IOLMaster measurement, and in another case, we calculated the secondary IOL power using the axial length of the partner eye. Corneal topography as well as the corneal aberrations were measured using a Pentacam AXL Wave (Oculus Optikgeräte GmbH, Wetzlar, Germany).

### Statistical analysis

In an intention-to-treat analysis statistical evaluation included dependent T-tests and Kruskal-Wallis tests for pre- and postoperative visual acuities. Intra- and postoperative complications were analysed using descriptive statistics. Chi-Square tests were used to test differences between the IOL haptic designs of the explanted IOLs (plate vs. three-piece) regarding intraoperative events and between the secondary IOLs (sulcus-fixated vs. iris-claw vs. capsular bag) regarding postoperative events. Independent T-tests were calculated between the refractive accuracy of the different groups of secondary IOL. The change of corneal astigmatism pre- and post-operative was evaluated using a calculation tool of the American Society of Cataract and Refractive Surgery (ASCRS)^8^. For calculation, a 180° correction was applied for all left eyes (180 – flat axis left eye) to account for the mirroring effect between the contralateral eyes^9^. Topography measurements as well as the changes in corneal higher order aberrations were compared using paired T-tests. Statistical analyses were achieved using SPSS software (version 29.0; IBM Corporation, Armonk, USA). A P value less than 0.05 was considered statistically significant. Bonferroni correction was applied to correct for multiple testing.

## Results

Thirty-five eyes undergoing IOL exchange surgery without ocular comorbidities apart from homogenous IOL calcification were included in this study. The mean age of the study population was 78 ± 8 years, with a male: female ratio of 2:3.

### Intraocular lenses

Calcification mainly affected plate-haptic-IOL designs (69% of all cases) from Oculentis (Oculentis Medical GmbH. Berlin, Germany) (37% of all cases) or Argonoptics (Argonoptics GmbH, Dreieich, Germany) (17% of all cases). In about half of the cases (46%) the manufacturer of the explanted IOL was unconfirmed. In 13 of the 35 eyes (37%), neodymium-doped yttrium aluminum garnet (Nd: YAG)-capsulotomy had been implemented prior to the IOL exchange. The main secondary lenses implanted were the retropupillary iris-fixated Artisan Aphakia (Ophtec, Groningen, Netherlands) (53%), and the sulcus-fixated AR40e (Johnson & Johnson Vision (J&J), Irvine, USA) (37%). In 3 cases (10%), in which the capsular support was intact after IOL removal, a preloaded IOL was placed into the capsular bag (Table 1). After explantation of the plate haptic primary IOL, in 13 cases an iris-fixated IOL and in 9 cases a sulcus fixated IOL was implanted. Capsular bag implantation was achieved in two cases. After explantation of the primary IOL with C-loop haptic, in 7 cases an iris-fixated IOL and in 3 cases a sulcus fixated IOL was implanted. In one case capsular bag implantation was possible.

**Table 1:** Characteristics of explanted and secondary intraocular lenses.

Explanted IOL			
Haptic design, N (%)	Plate, 24 (69%)		C-loop, 11 (31%)
Manufacturer (%)	Oculentis (46%), unknown (54%)		Argonoptix (64%), unknown (36%)
Secondary IOL			
Fixation site, N (%)	Iris-fixated, 20 (57%)	Sulcus-fixated, 12 (34%)	Capsular-bag, 3 (9%)
Manufacturer/ Model (%)	Ophtec/ Artisan Aphakia (100%)	J&J/ AR40e (100%)	J&J/ DIB00, Hoya/ XY1-EM, Kowa/ CP2.2R (one each)

*IOL* Intraocular lens, *N* total number, %, percentage.

### Visual acuity

CDVA improved from 0.16 ± 0.14 logMAR to 0.07 ± 0.14 logMAR (*p* = 0.04). Eyes treated with an iris-fixated IOL yielded 0.09 ± 0.16 logMAR, eyes with a sulcus-fixated IOL 0.04 ± 0.1 logMAR, and those with a capsular bag IOL 0.09 ± 0.16 logMAR (*p* = 0.67).

### Refractive outcome

**Fig. 1:**
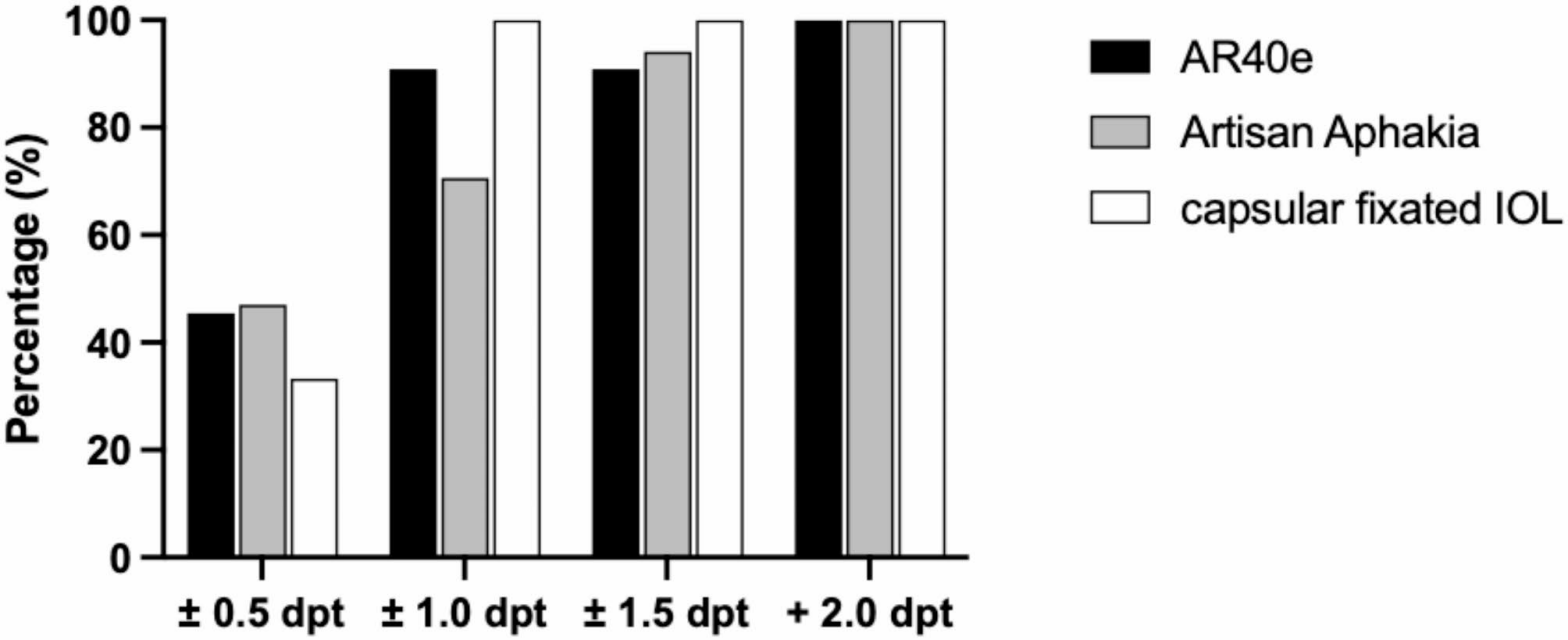
Difference between postoperative spherical equivalent and target refraction of the IOLMaster, grouped by secondary IOL. Percentages (%); differences in Diopter.

Emmetropia was chosen as target refraction in all but two cases that were aimed for minus 2.5 D. The mean deviation from target refraction for the Artisan Aphakia, the AR40e and the capsular bag fixated IOLs were + 0.53 ± 0.62, − 0.26 ± 0.78, − 0.43 ± 0,52 D, respectively. In about half of the cases the difference between the spherical equivalent and the target refraction was within ± 0.5 D (Artisan Aphakia: 47%, AR40e: 45%, Capsular: 33%) and in the majority within ± 1.0 D (Artisan Aphakia: 71%, AR40e: 90%, Capsular: 100%) (Fig. 1).

### Astigmatism

**Fig. 2:**
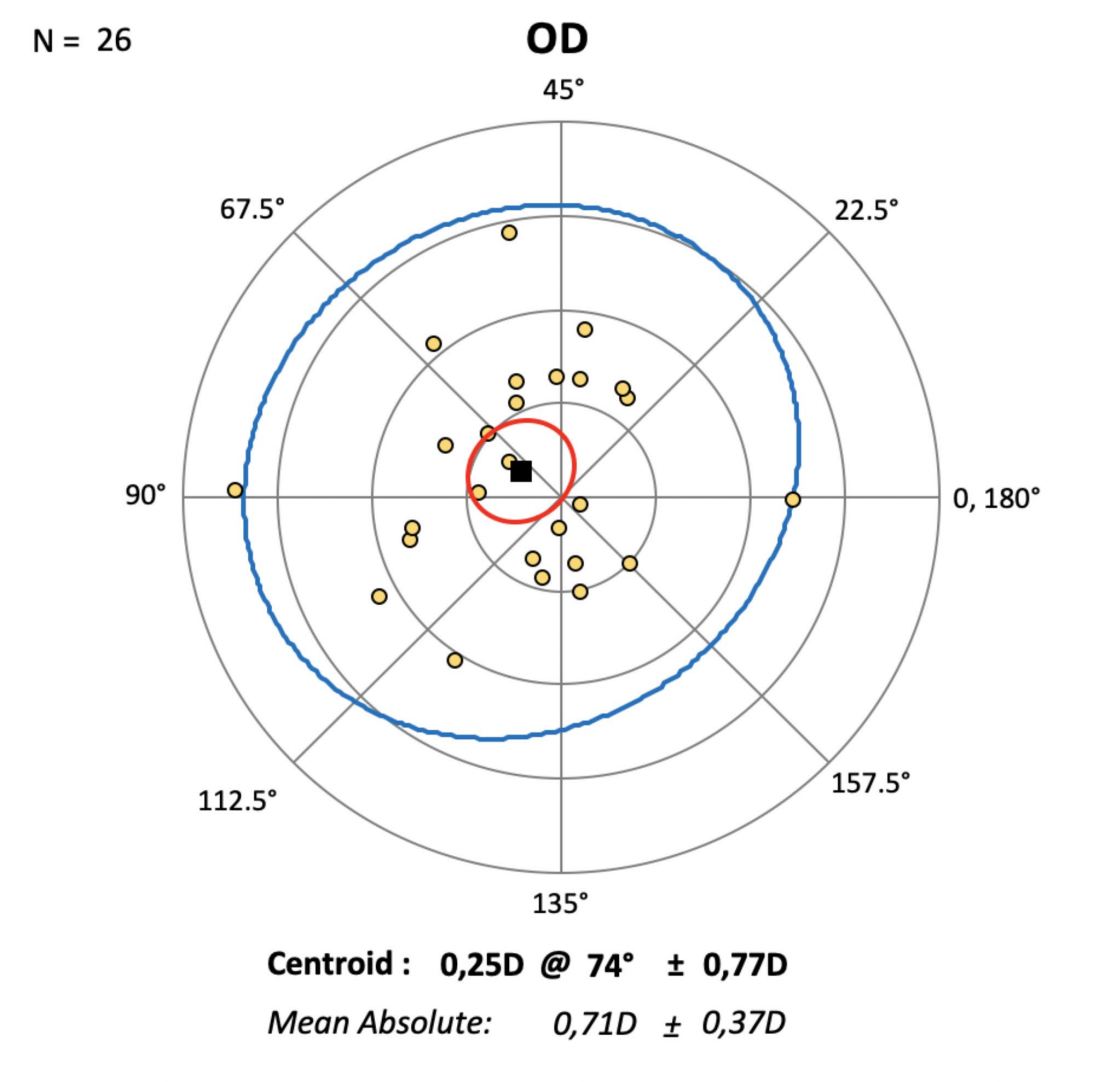
Individual changes in corneal astigmatism after IOL exchange. Centroid (black square, Diopter (D) at axis (degree). 95% confidence ellipse of the centroid (red circle). 95% confidence ellipse of the dataset (blue circle). Each ring = 0.50 Diopter.

**Fig. 3:**
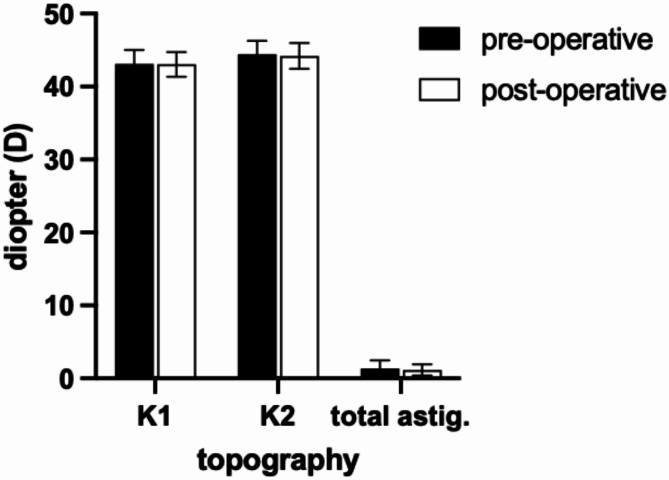
Corneal topography pre- and postoperative for K1 and K2 value as well as the total corneal astigmatism. Mean values in Diopter (D) with standard deviation.

The mean astigmatism before the secondary surgery was 1.27 D ± 1.06 D. The corneal astigmatism differed postoperative by 0.25 D at 74° ± 0.77 D. The 95% confidence interval of the centroid was outside the 0 D, which implies a small but statistically significant effect (Fig. 2). The keratometry K1 and K2 as well as the overall astigmatism did not show statistically significant differences (K1: *p* = 0.34, K2 *p* = 0.19, total astigmatism: *p* = 0.85) (Fig. 3).

### Corneal lower and higher order aberrations

**Fig. 4:**
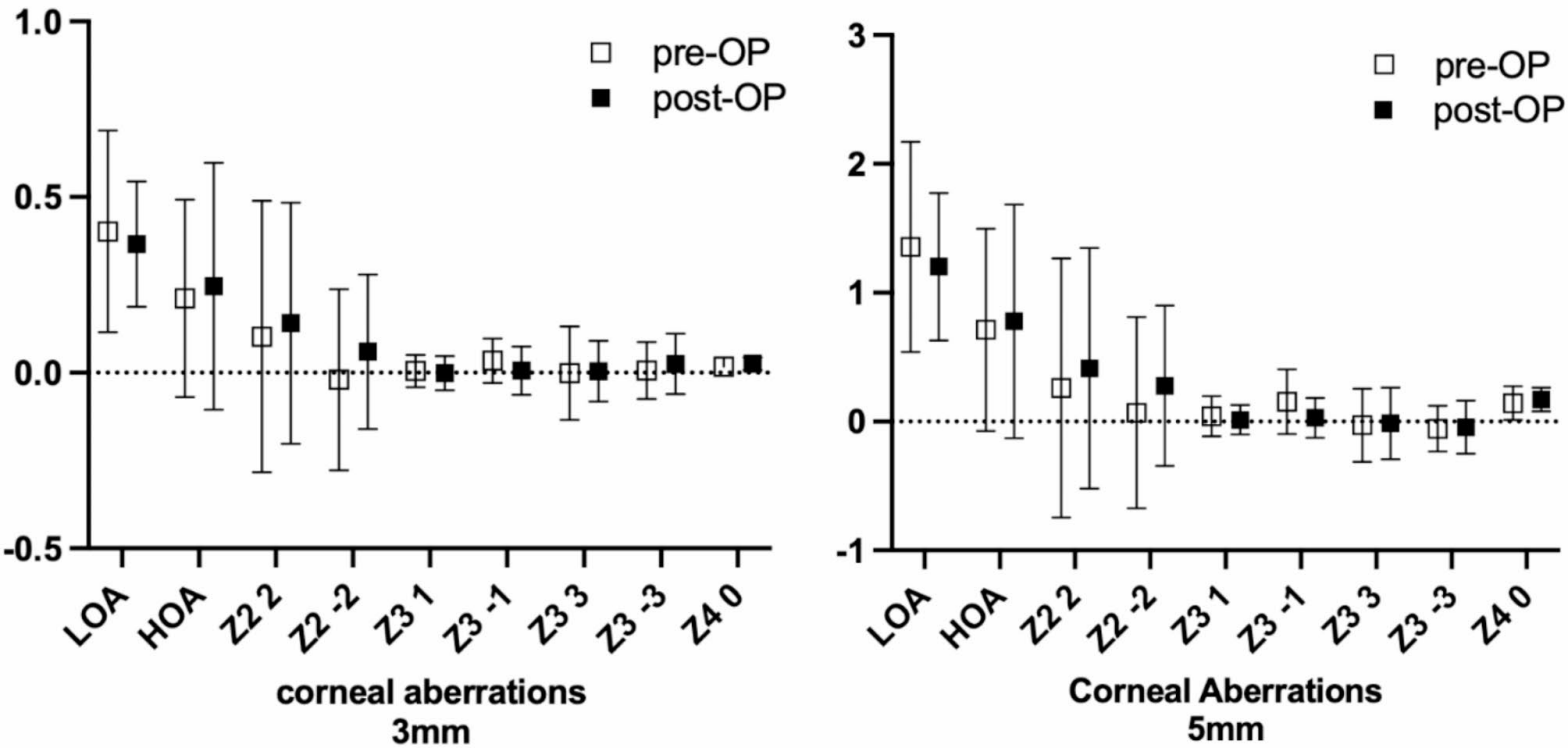
Corneal lower and higher order aberrations (LOA/ HOA), horizontal and vertical astigmatism (Z2 2, Z2 -2), coma (Z3 1, Z3 -1), trefoil (Z3 3, Z3 -3) and spherical aberrations (Z4 0) pre- and post-operative for the 3 mm and 5 mm zone. Mean values in Diopter (D) with standard deviation.

In the 3- and 5-mm zones the corneal total lower and higher order aberrations (LOA/ HOA) as well as horizontal and vertical astigmatism, coma, trefoil and spherical aberrations pre- and postoperative did not differ statistically significant (Table 2; Fig. 4).

**Table 2:** Corneal lower and higher order aberrations pre- and postoperative under different pupil conditions.

	3 mm pupil			5 mm pupil		
	Preoperative	Postoperative	P*	Preoperative	Postoperative	P*
RMS LOA	0.40 ± 0.29	0.37 ± 0.18	*> 0.05*	1.36 ± 0.82	1.20 ± 0.57	*> 0.05*
RMS HOA	0.21 ± 0.28	0.25 ± 0.35	*> 0.05*	0.71 ± 0.79	0.77 ± 0.90	*> 0.05*
Z2 2	0.10 ± 0.39	0.14 ± 0.34	*> 0.05*	0.26 ± 1.01	0.41 ± 0.93	*> 0.05*
Z2 -2	− 0.02 ± 0.26	0.06 ± 0.22	*> 0.05*	0.07 ± 0.74	0.28 ± 0.62	*> 0.05*
Z3 1	0.005 ± 0.05	− 0.001 ± 0.05	*> 0.05*	0.04 ± 0.15	0.01 ± 0.11	*> 0.05*
Z3 -1	0.034 ± 0.06	0.006 ± 0.07	*> 0.05*	0.15 ± 0.25	0.03 ± 0.15	*> 0.05*
Z3 3	− 0.001 ± 0.13	0.004 ± 0.09	*> 0.05*	− 0.03 ± 0.28	− 0.01 ± 0.28	*> 0.05*
Z3 -3	0.007 ± 0.08	0.025 ± 0.09	*> 0.05*	− 0.06 ± 0.18	− 0.04 ± 0.21	*> 0.05*
Z4 0	0.02 ± 0.03	0.025 ± 0.02	*> 0.05*	0.14 ± 0.13	0.17 ± 0.09	*> 0.05*

*RMS* root mean square, Corneal lower and higher order aberrations (LOA/HOA); horizontal and vertical astigmatism (Z2 2, Z2 -2); coma (Z3 1, Z3 -1); trefoil (Z3 3, Z3 -3) and spherical aberrations (Z4 0); *paired T-tests.

### Intra- and postoperative events

In 81% of all cases, an anterior vitrectomy was required. In most of the cases the surgeon decided to perform an additional posterior vitrectomy (78%). In 45% of all cases capsular defects or zonular instability was described. In 37% of cases the posterior capsular had already been opened by Nd: YAG-capsulotomy prior to the IOL exchange. No statistically significant differences were found regarding the haptic design of the explanted IOL and intraoperative events (*p* = 0.74) (Table 3). Postoperatively, several complications (mostly mild) were described: one third of eyes showed temporary anterior chamber bleeding that resolved without postoperative sequelae. Iris-related changes, such as iris stromal defects were present in one quarter of the postoperative eyes. A minority had other mild complications like transient corneal edema, subconjunctival hemorrhage (hyposphagma) or a corneal erosion. More severe complications included increased transient intraocular pressure (IOP) (11%) that resolved under topical antiglaucomatous eye drops. One case, where the IOP increase was due to an anterior chamber bleeding, required surgical clearing. In three cases, there was a mild vitreous bleeding, that resolved spontaneously. In two cases, the secondary IOL showed decentration postoperatively. In one of these cases, the IOL was repositioned surgically, using optic capture to secure its position (Table 4). There were no differences between the primary IOL haptics design (plate vs. C-loop) and postoperative complications, including anterior chamber bleeding (*p* = 0.58) and postoperative iris changes (*p* = 0.60).

**Table 3:** Intraoperative events and findings separated by the type of explanted IOL.

	Plate haptic	C-loop	p*
Capsular defects or zonular instability	11 (33)	4 (12)	> 0.05
Anterior vitrectomy	19 (57)	8 (24)	> 0.05
Posterior vitrectomy	17 (51)	9 (27)	> 0.05

Total number (N) and percentage (%); IOL, intraocular lens; *Chi-Square Tests

**Table 4:** Postoperative events.

Severity	Event	Type of secondary IOL, total number (*N*) and percentage (%)		
		Iris-fixated	Sulcus-fixated	Capsular bag IOL
Mild	Anterior chamber bleeding	7 (21)	5 (15)	0 (0)
	Iris findings (iris stromal defects/ transillumination/ distorted pupil)	9 (27)	0 (0)	0 (0)
	Corneal edema	3 (9)	1 (3)	0 (0)
	Vitreous bleeding	0 (0)	2 (6)	0 (0)
	Hyposphagma	1 (3)	0 (0)	1 (3)
	Corneal erosion	1 (3)	0 (0)	0 (0)
Moderate to severe	Increased IOP	0 (0)	2 (6)	0 (0)
	IOL-subluxation	1 (3)	1 (3)	0 (0)
	Macular edema	1 (3)	0 (0)	0 (0)
	Re-surgery	2 (6)	0 (0)	0 (0)
	Corneal decompensation	1 (3)	0 (0)	0 (0)

*IOP* intraocular pressure, *IOL* intraocular lens.

## Discussion

In this prospective, observational clinical study of IOL exchange through a sclero-corneal tunnel, we analysed the impact of the surgery on the refractive outcome and changes in corneal aberrations. Furthermore, intra- and postoperative events were recorded. While a few previous observational studies on IOL exchange achieved a standardized investigation of functional outcomes and complications, these studies usually investigate a small number of eyes and do not exclude comorbidities, which we consider has the potential to bias results. Our cohort comprised eyes without any ocular disease other than homogeneous IOL calcification. There was an improvement in visual acuity of approximately one line three months after surgery. The amount of visual recovery after this invasive surgery is in concordance with the published literature: in a study by Fernandez-Buenaga et al., CDVA increased from 0.49 ± 0.31 logMAR to 0.18 ± 0.22 logMAR^2^. Patients from our study achieved a better postoperative CDVA of 0.07 ± 0.14 logMAR. However, our cohort also showed better preoperative visual acuity which can be explained by the inclusion criteria of our study that did not allow ophthalmic comorbidities apart from the IOL opacity. Similar to our approach, Mäker et al. only included eyes with IOL calcifications without comorbidities in their study on IOL exchange^3^. Visual acuity in their cohort improved from 0.42 ± 0.32 to 0.25 ± 0.28 logMAR^3^. A study by Stewart et al. showed a better visual acuity of − 0.02 ± 0.10 logMAR after IOL-exchange after a recovery time of 6.9 ± 6.0 months^10^. A recent IRIS Registry study on IOL exchange revealed that the postoperative visual acuity depends on the preoperative visual acuity, with poorer preoperative CDVA predicting worse postoperative values^11^. The authors linked poor preoperative values to more ocular comorbidities^11^. Our study confirms that patients without comorbidities with fairly good preoperative visual acuity achieve good postoperative results after IOL exchange surgery.

In about half of our cases, the target refraction was within a ± 0.5 D range. In 70% of cases implanted with a retropupillary iris-claw lens, up to 90% of cases with a sulcus-fixated IOL, and in all cases with an in-the-capsula-bag IOL the target refraction was within ± 1 diopters. Reasons for a deviation from the planned target refraction are errors in the measurement of axial length and keratometry and the preoperative estimation of the postoperative effective lens position. A previous study compared axial length measurements before the initial cataract surgery and before the IOL exchange. The authors reported that axial length measurements were 0.3–0.4 mm shorter before IOL exchange than before cataract surgery^12^. Furthermore, positioning of the IOL might be more difficult after IOL exchange, e.g. due to the instability of the capsular support structures. One major factor that can influence achieving the intended target refraction lies in the difficulty of measuring the axial length in eyes with opacified lenses. When opacification prevents an optical biometer such as the IOLMaster, then A-scan biometry is needed to obtain the axial length. In our study, the IOLMaster could not measure the axial length in five eyes. We used A-scan biometry in three of these eyes. In one of the five, the axial length of the partner eye was used and in the fifth, we used the patient’s earlier biometry measurement. However, getting a valid measurement with the A-scan in such cases is dependent on the experience of the user and more prone to errors than modern optical biometry. Using the axial length from the partner eye or from earlier measurements can also be prone to errors.

In contrast, keratometry plays a minor role in achieving the intended target refraction in this cohort. One former study showed that astigmatism before cataract surgery and before IOL exchange does not differ significantly^12^. Our data adds that corneal astigmatism also does not change significantly after IOL exchange through a sclero-corneal tunnel. Our results still show a small statistically significant change. However, the value from our study (0.25 D ± 0.77 D) is low. In the study of Eum et al. they operated by either implanting a scleral fixation IOL or by refixing the primary IOL. They reported a SIA at 3 months of 1.29 D ± 0.46 D in eyes that underwent IOL exchange and in the cases of IOL refixation the SIA was 0.79 D ± 0.41 D^5^. Kubaloglu et al. described a mean SIA of 0.7D ± 0.24 D after IOL exchange using a clear corneal incision and cutting the IOL in half for explantation, which they rated as higher than standard cataract surgery but still acceptable^6^. A study by Cao et al. evaluated the SIA in uncomplicated clear corneal incision (CCI) femtosecond laser-assisted cataract surgery. At 6 months post-surgery, the SIA was 0.37 D ± 0.25 D for a superior incision^13^. Compared to our results, the change in astigmatism after IOL exchange though a scleral-corneal tunnel and the SIA after superior CCI cataract surgery are similar. These data show that IOL exchange through a scleral-corneal incision does not impact the astigmatism change more than uncomplicated cataract surgery. In addition to these findings, Dan at al. found no significant difference in SIA between limbal tunnel incision (0.33 D ± 0.32D) and CCI (0.24 D ± 0.14 D) after implantation of an ICL 6 months after surgery^14^. Therefore, using a scleral-corneal tunnel with an iris-, sulcus- or capsular bag fixation might be a good method for IOL exchange, keeping the impact of surgery on the refractive outcome to a minimum. Likewise, our data suggests that IOL exchange through a scleral-corneal tunnel does not have a strong impact on aberrations of higher order, which was not studied before.

In our group, 80% of surgeries needed anterior vitrectomy. In almost all cases (77%) the eyes underwent additional posterior vitrectomy. This rate was higher compared to other studies. In the study by Mäker et al. mentioned above, only 40% of patients underwent anterior vitrectomy^3^. In the study by Stewart et al. even less eyes (9 eyes, 24%) were treated with anterior vitrectomy and two (5%) eyes with posterior vitrectomy^3,10^. A high rate of prior Nd: YAG-capsulotomies of our cohort can explain the rather high rate of vitrectomies. In most cases, the lack of secure capsular support prevented IOL implantation in the capsular bag. Therefore, we implanted mainly iris- and sulcus-fixated IOLs. Despite the lower rate of anterior vitrectomies in the study by Märker et al., the authors also placed the secondary IOL in the capsular bag in only 4.2%^3^. Instead, similar to our approach, in most of the cases the IOL was placed in the ciliary sulcus (72.9%) or as an iris-claw lens (22.9%). Stewart et al. were able to perform in-the-bag implantations in about 35% of cases^10^. However, sulcus-fixation was also the most common approach (59.5%, iris-fixation: 5%)^10^. Likewise, a study by Kim et al. showed a low number of cases with capsular instability (9.6%) and the need for anterior vitrectomy (13.5%). As a result, in-the-bag implantation of the secondary IOL was possible in one quarter of cases, with implantation in the sulcus in the rest (75%)^2,3,12^. When capsular bag support is absent, the differences between the types of secondary IOLs used can also be explained by the study’s geography as iris-fixated lenses are not available in every country, then alternatives are used, such as anterior chamber or sclero fixation In a United States-based study analyzing IOL exchange, secondary IOL positions were mainly in the capsular bag (43.1%), followed by anterior chamber (25.7%) and sulcus fixation (22%)^15^. No retropupillary iris-fixated IOLs were used, since these IOLs are not approved for sale on the USA.

When postoperative complications occurred, they were minor, resolved spontaneously and rarely required further intervention. Furthermore, intra- and postoperative complications were not influenced by the haptic design of the explanted lens. We classified only a few complications as moderate to severe. Two eyes (6%) developed a cystoid macular edema (CME) postoperatively, which is in line with the literature, where the incidence of CME in patients following IOL exchange due to IOL dislocation ranges between 0 and 24%^16^. In our study, no retinal detachment was reported during the follow up period. One explanation could be that almost all eyes that underwent anterior vitrectomy also underwent posterior vitrectomy for safety reasons. Previous studies suggest that the risk for retinal detachment is lower in eyes with posterior vitrectomy compared to anterior vitrectomy only after cataract surgery with capsular rupture^17^. Nevertheless, we cannot rule out the development of retinal detachment after a longer postoperative period. Similarly, we did not experience any case with postoperative formation of an epiretinal membrane. The IRIS registry study, however, found this to be a common postoperative complication one year after IOL exchange^11^. Hence, some eyes might develop an epiretinal membrane beyond the period of our investigation. Another limitation of this study is that we focused on IOL calcifications as the reason for IOL exchange. Further research might reveal that the results are different for other conditions, such as IOL dislocation. We decided to study an eye-healthy cohort to have a homogeneous group and thus minimize the influence of confounding factors. However, additional factors, such as corneal healing response, ocular surface, tear film quality, and subclinical corneal edema, could have influenced the results. Despite these considerations, our results did not indicate significant changes in HOAs, although we acknowledge the observed trend toward increased aberrations.

In conclusion, visual recovery after IOL exchange is good in eyes without additional comorbidities. Target refraction is achieved in most cases and IOL exchange does not seem to have a major effect on corneal topography or aberrations of higher order. Mild complications such as anterior chamber bleeding and iris defects can occur; but more severe complications are rare. The explanted IOL’s haptic design does not influence the occurrence of intra- or postoperative complications.

## Data Availability

The datasets used and analysed during the current study available from the corresponding author on reasonable request.
